# Duodenal perforation in the excluded segment: twenty-five years post-Roux-en-Y gastric bypass surgery: a case report

**DOI:** 10.1093/jscr/rjaf856

**Published:** 2025-10-24

**Authors:** Neha Aftab, Sara N Nesheiwat, Carlos Martinez Elizondo, Sean Lewis, Kristoffer Wong, Gul R Sachwani-Daswani

**Affiliations:** Department of Trauma and Acute Care Surgery, Hurley Medical Center, 1 Hurley Plaza, Flint, MI 48503, United States; Department of Surgery, Michigan State University College of Human Medicine, 200 E 1st St, Flint, MI 48502, United States; Department of Surgery, Henry Ford Genesys Hospital, 1 Genesys Pkwy, Grand Blanc, MI 48439, United States; Department of Surgery, Henry Ford Hospital Macomb, 19 Mile Rd, Clinton Township, MI 48038, United States; Department of Trauma and Acute Care Surgery, Hurley Medical Center, 1 Hurley Plaza, Flint, MI 48503, United States; Department of Surgery, Michigan State University College of Human Medicine, 200 E 1st St, Flint, MI 48502, United States; Department of Trauma and Acute Care Surgery, Hurley Medical Center, 1 Hurley Plaza, Flint, MI 48503, United States; Department of Surgery, Michigan State University College of Human Medicine, 200 E 1st St, Flint, MI 48502, United States; Department of Trauma and Acute Care Surgery, Hurley Medical Center, 1 Hurley Plaza, Flint, MI 48503, United States; Department of Surgery, Michigan State University College of Human Medicine, 200 E 1st St, Flint, MI 48502, United States

**Keywords:** bariatric surgery, bariatric surgery complications, duodenal perforation, duodenal ulcer, Roux-en-Y gastric bypass, RYGB

## Abstract

Duodenal perforations after Roux-en-Y gastric bypass surgery are a rare, but potentially life-threatening complication that poses significant challenges in diagnosis and treatment due to altered anatomy and several other risk factors such as nonsteroidal anti-inflammatory drugs, alcohol, and *Helicobacter pylori* infection. We present a case of a 70-year-old female status post Roux-en-Y gastric bypass for weight loss 25 years ago with chief complaint of sharp stabbing abdominal pain; she was later found to have a was later found to have a perforation of the biliopancreatic limb, specifically at D3. Early surgical intervention remains crucial in these patients for improving outcomes. Clinicians should be aware of diagnostic delays reported in the literature; and early surgical exploration (i.e. laparoscopy ± conversion to exploratory laparotomy) should be considered when clinical suspicion is high.

## Introduction

Roux-en-Y gastric bypass (RYGB) is one of the most common bariatric surgeries performed, having been utilized in practice for over 30 years. RYGB surgery provides effective and sustainable weight loss with clinically and statistically proven positive effects on obesity-related comorbidities [1]. With this longstanding history, the possible complications associated with this operation and their incidences have been well documented.

Postoperative complications are classified as early or late and include both physical and metabolic issues [2]. Complications such as Dumping Syndrome, Blind Pouch Syndrome, internal hernias, anastomotic leaks, and marginal ulcers should be considered in the differential diagnosis. Although rare, perforation is among the most technically challenging complications to manage surgically.

Perforations in RYGB patients are often broken down by location. Early perforations are usually associated with an anastomosis breakdown or staple line leak, with an incidence ranging from 0.4% to 0.6% [3]. A later source of perforation in these patients tends to be marginal ulcer perforations, which are usually near the gastrojejunal anastomosis with an incidence of 1.0%–2.0% [4]. Gastric remnant perforation is another late complication, with an incidence rate of 0.12% to 0.84% [5].

Due to the severity of perforation in RYGB patients, prompt diagnosis is essential for effective surgical management. This case highlights the rare occurrence of a D3 perforation 25 years after RYGB and underscores the value of learning from such uncommon presentations.

## Case presentation

**Figure 1:**
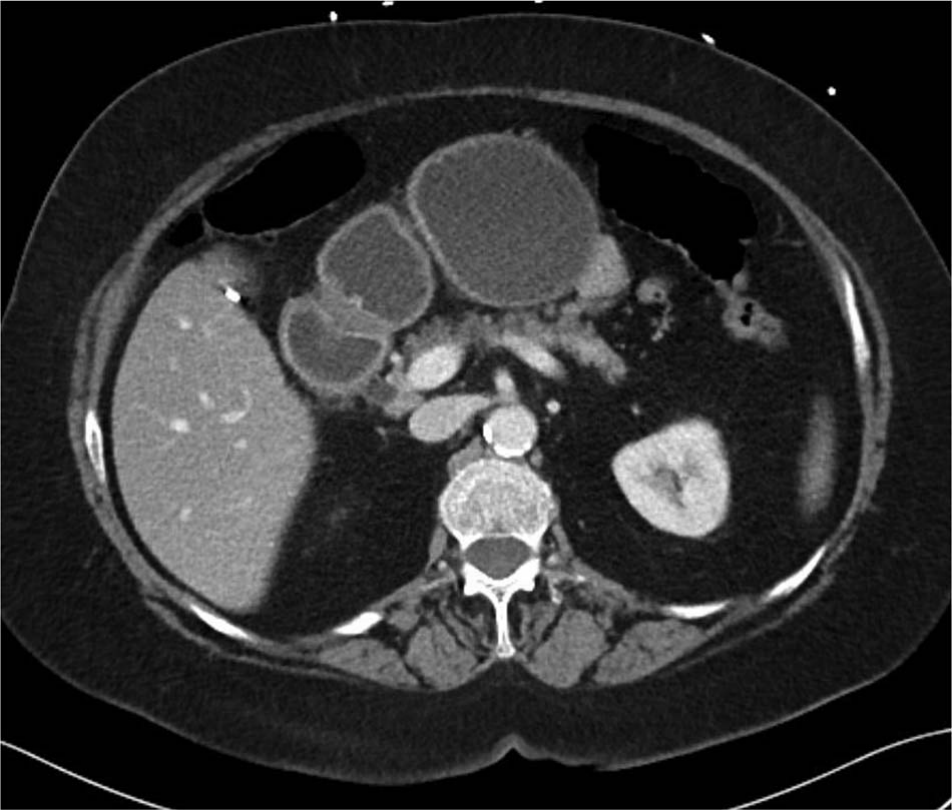
CT scan A/P of the abdomen and pelvis with IV contrast on presentation showing moderately dilated gastric remnant with thickening of the pylorus and proximal duodenum.

**Figure 2:**
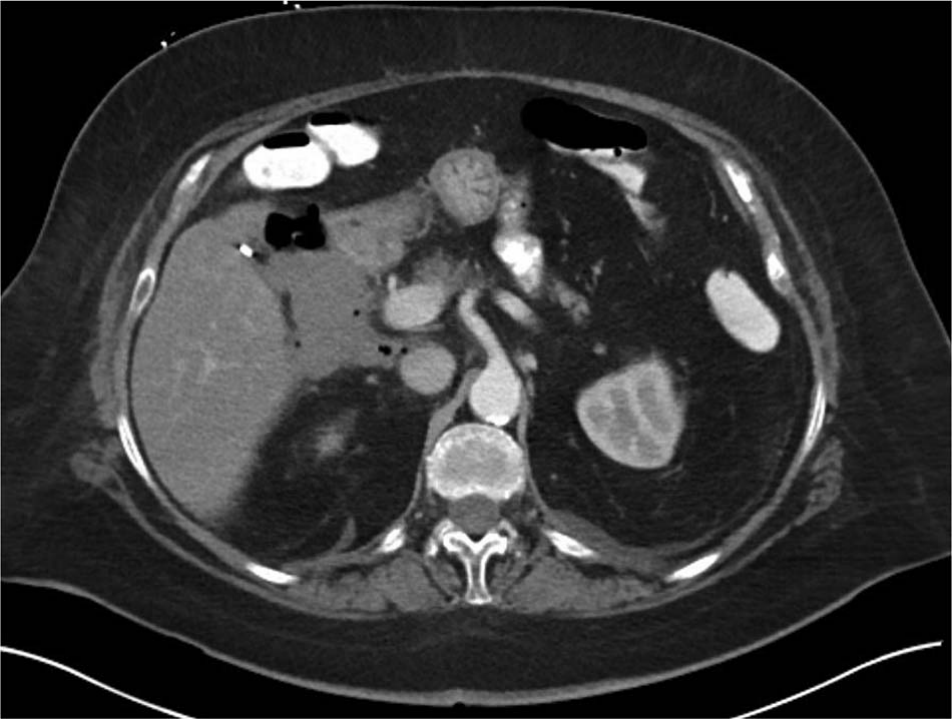
Hospital Day 4 CT scan of the abdomen and pelvis A/P with oral and intravenous contrast demonstrating scattered pneumoperitoneum and free fluid by lateral duodenum.

**Figure 3:**
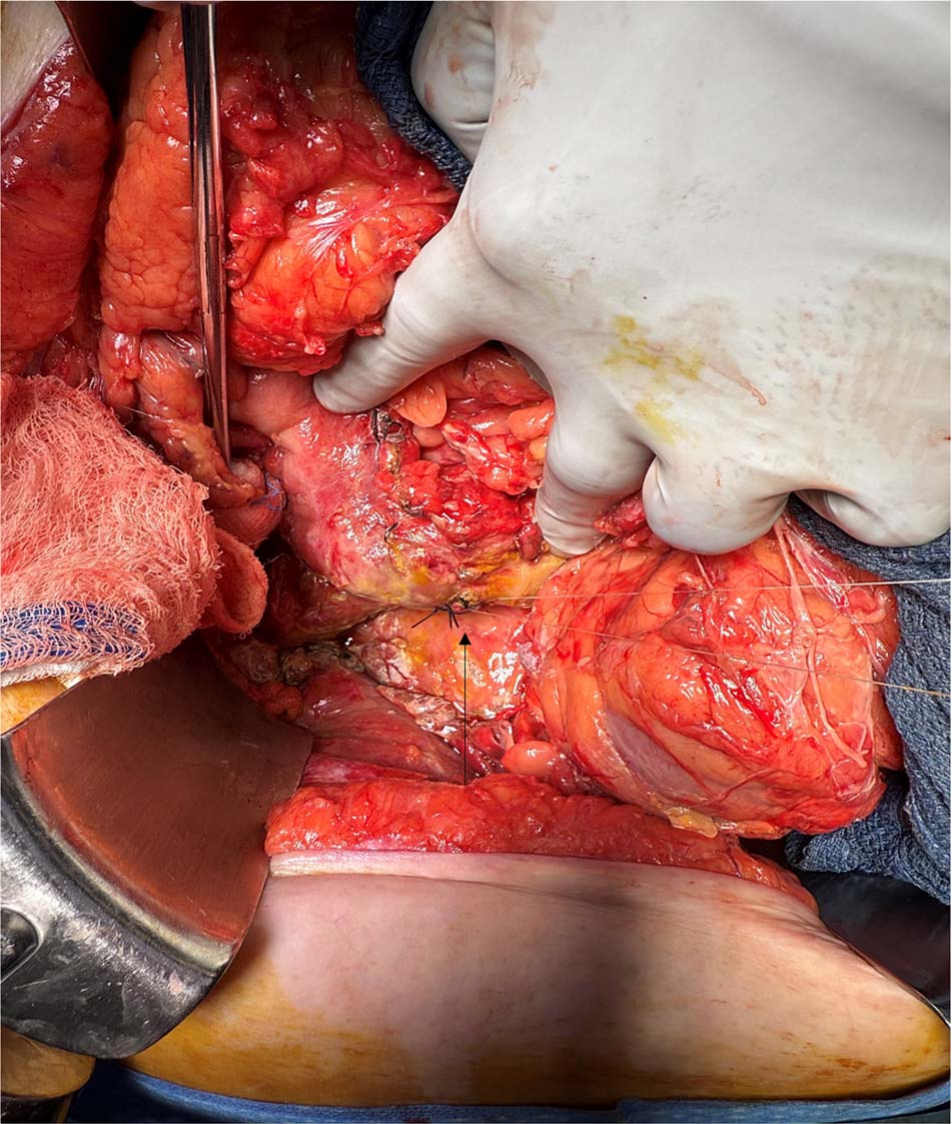
Intra-operative image showing primary repair of D3 duodenal perforation.

**Figure 4:**
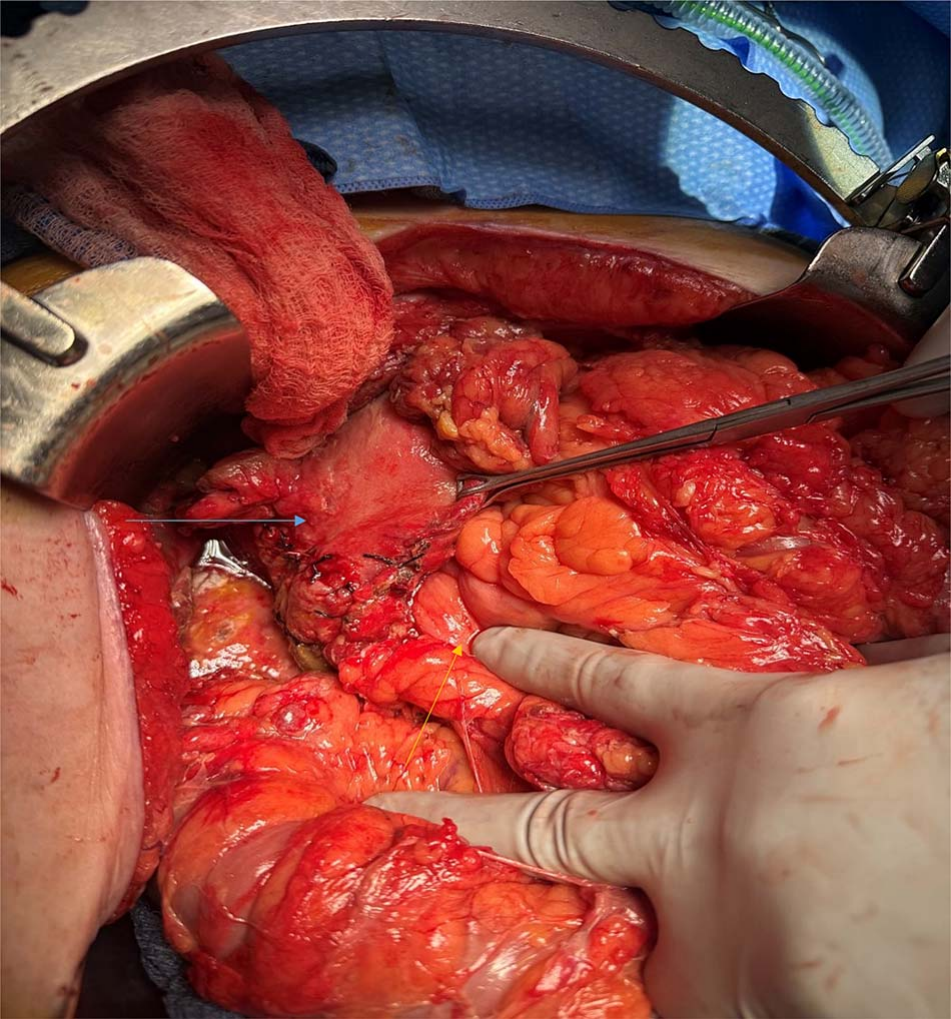
Intra-operative image showing pyloroplasty and omental patch repair.

A 70-year-old woman with obesity, non-insulin-dependent diabetes, gastroesophageal reflux disease (GERD), cholecystectomy, and prior RYGB presented to the ED with acute chest and lower abdominal pain. The pain began 1 day earlier and initially responded to Ibuprofen, which she had been taking 4–6 times daily for musculoskeletal discomfort. Her pain worsened, radiating to her back, and was accompanied by belching and vomiting with dry heaving. She denied trauma or changes in bowel habits. Initial CT (Fig. 1) imaging showed a moderate hyperdense area around the duodenum, distension of the afferent limb and excluded stomach, and expected postoperative changes. She was admitted for conservative management. Four days later, a repeat CT (Fig. 2) was performed due to persistent symptoms, revealing free fluid in the intraperitoneal and retroperitoneal spaces, raising concern for duodenal perforation. She developed lethargy and diffuse abdominal tenderness and was taken to the operating room. Exploratory laparotomy with peritoneal washout, enterolysis, partial omentectomy was performed. It revealed two 1 cm posterolateral perforations of D3, which were repaired with an omental pedicle flap. The Roux limb was healthy, the jejunojejunostomy was dilated but patent, and the biliopancreatic limb was intact. Due to high vasopressor requirements, a temporary abdominal closure device was placed, and she was transferred to the surgical ICU. After stabilization, a second-look laparotomy was performed the next day to reinforce the duodenal closure, place a gastrostomy tube for drainage, and insert a J-tube for feeding (Figs 3 and 4). The abdominal wall was closed. On postoperative day 2, a CT with contrast showed extravasation from the duodenal repair site. Interventional radiology placed a transhepatic biliary drain to divert bile and support duodenal patch healing. *Helicobacter pylori* antigen was negative. Her condition improved, and she was started on an enteral and slow oral feeding regimen. However, at this juncture, the patient declined further treatment and requested comfort care measures.

## Discussion

The rising prevalence of RYGB among individuals with obesity is attributed to sustained weight loss and the resolution of comorbidities such as diabetes and dyslipidemia, with an acceptable complication profile. After 12 months, patients typically experience a 70% reduction in excess body weight [6]. Early complications, including anastomotic leak, fistula, ulcer, and stenosis, occur more frequently than long-term complications. Common long-term complications include GERD, dumping syndrome, and cholecystitis [7].

Despite favorable outcomes, the detection and management of late complications such as duodenal perforation remain challenging. Compared to marginal ulcers, whose incidence, presentation, and management has been well documented, enough evidence regarding ulceration and perforation in excluded segments (i.e. duodenum), particularly their diagnosis and treatment is limited to case reports/series [8].

Plitzko *et al*. conducted a literature review of 54 patients described in case reports and case series, demonstrating that the most frequent presentations of peptic ulcer disease in this population are bleeding or perforation. The review also indicated that the time to presentation ranged from 2.5 months to twenty years following RYGB [8].

Our patient developed a duodenal perforation 25 years after RYGB. This case highlights the diagnostic challenges posed by altered anatomy and initial vague symptomatology. The pathophysiology of duodenal perforation following RYGB remains incompletely understood, although several risk factors have been identified. The gastric remnant retains acidity due to unbuffered acid secretion and reduced gastrin levels, as food bypasses this region and neutralization does not occur. While most patients are prescribed proton pump inhibitors, altered gastric mucosa may impair drug absorption. Additional risk factors include *H. pylori* infection, smoking, nonsteroidal anti-inflammatory drug (NSAID) use, and alcohol consumption [8, 9]. In our patient, increased NSAID intake for knee osteoarthritis was the only risk factor identified beyond anatomical alterations.

Diagnosing duodenal perforation in post-RYGB patients is challenging because the altered anatomy prevents air from entering the duodenum, so pneumoperitoneum, a sign of perforation, is often absent or delayed. CT scans may be negative; however, the absence of free air or increased fluid should prompt suspicion for perforation, and surgical intervention should be considered at a lower threshold. In our case, the initial CT scan was negative, but a repeat scan revealed free fluid in the intraperitoneal and retroperitoneal spaces.

Timely surgical intervention is crucial for improving outcomes in these cases. Surgeons should recognize the limitations of conventional imaging in post-RYGB patients and maintain a lower threshold for diagnostic laparoscopy. Given the diagnostic delays reported in the literature, documenting cases such as ours is essential, and larger studies are necessary to establish guidelines for the diagnosis and management of this complication in this patient population.
